# Supplementary material for: Loss of dihydroceramide desaturase drives neurodegeneration by disrupting endoplasmic reticulum and lipid droplet homeostasis in glial cells
Source: eLife. 2025 Aug 27;13:RP99344. doi: 10.7554/eLife.99344 (PMC12387754; doi:10.7554/eLife.99344)
Supplement: Figure 1—figure supplement 3—source data 1. [file elife-99344-fig1-figsupp3-data1.docx]

All lines that were tested are listed below:

**Mi{GFP[E.3xP3]=ET1} transgenes tested:**

y[1] w[67c23]; Mi{GFP[E.3xP3]=ET1}MB00051: BDSC-22685

y[1] w[67c23]; Mi{GFP[E.3xP3]=ET1}MB00056: BDSC-22686

w[1118]; Mi{GFP[E.3xP3]=ET1}Ppn[MB07666]: BDSC 25277

**Tl{GFP[3xP3.cLa]=CRIMIC.TG4} transgenes tested:**

y[1] w[*]; TI{GFP[3xP3.cLa]=CRIMIC.TG4.0}mfr[CR70821-TG4.0]: BDSC 98524

y[1] w[*]; TI{GFP[3xP3.cLa]=CRIMIC.TG4.0}lace[CR70094-TG4.0]/SM6: BDSC 92702

y[1] w[*] TI{GFP[3xP3.cLa]=CRIMIC.TG4.2}schlank[CR01622-TG4.2] Josd[CR01622-TG4.2-X]/FM7: BDSC 91266

y[1] w[*]; TI{GFP[3xP3.cLa]=CRIMIC.TG4.0}bwa[CR02807-TG4.0]/SM6a; BDSC 97620

y[1] w[*]; TI{GFP[3xP3.cLa]=CRIMIC.TG4.2}ifc[CR70115-TG4.2]/SM6a; BDSC 92710

y[1] w[*]; TI{GFP[3xP3.cLa]=CRIMIC.TG4.1}ghi[CR01805-TG4.1]/TM3 ; BDSC 92656

**M{3xP3-RFP.attp} transgenes tested**

M{3xP3-RFP.attP}ZH-2A: Derived from BDSC 24480

M{3xP3-RFP.attP’}ZH-22A: Derived from BDSC 24481

M{3xP3-RFP.attP’}ZH-51C: Derived from BDSC 24482

M{3xP3-RFP.attP}ZH-51D: Derived from BDSC 24483
