# Supplementary material for: Loss of dihydroceramide desaturase drives neurodegeneration by disrupting endoplasmic reticulum and lipid droplet homeostasis in glial cells
Source: eLife. 2025 Aug 27;13:RP99344. doi: 10.7554/eLife.99344 (PMC12387754; doi:10.7554/eLife.99344)
Supplement: Supplementary file 1. [file elife-99344-supp1.docx]

**Supplementary File 1. Genotype of flies in Figures 2 & 3**

| **Figure** | **Glial subtype** | **Group** | **Genotype** |
| --- | --- | --- | --- |
| Figure 2 (a) | Cortex glia | Wild type | *GMR 54HO2-GAL4/10XUAS-IVS-myr-GFP* |
| Figure 2 (a’) | Cortex glia | *ifc^-/-^* mutant | *ifc-KO/ifc^js3^; GMR 54HO2-GAL4/10XUAS-*  *IVS-myr-GFP* |
| Figure 2 (b) | Ensheathing glia | Wild type | *GMR 56F03-GAL4/10XUAS-IVS-myr-GFP* |
| Figure 2 (b’) | Ensheathing glia | *ifc^-/-^* mutant | *ifc-KO/ifc^js3^; GMR 56F03-GAL4/10XUAS- IVS-myr-GFP* |
| Figure 2 (c) | Astrocyte-like  glia | Wild type | *GMR86EO1-GAL4/10XUAS-IVS-myr-GFP* |
| Figure 2 (c’) | Astrocyte-like  glia | *ifc^-/-^* mutant | *ifc-KO/ifc^js3^; GMR86EO1-GAL4/10XUAS-*  *IVS-myr-GFP* |
| Figure 2 (d) | Subperineurial  glia | Wild type | *GMR 54C07-GAL4/10XUAS-IVS-myr-GFP* |
| Figure 2 (d’) | Subperineurial  glia | *ifc^-/-^* mutant | *ifc-KO/ifc^js3^; GMR 54C07-GAL4/10XUAS-*  *IVS-myr-GFP* |
| Figure 2 (e) | Perineurial glia | Wild type | *GMR 85G01-GAL4/10XUAS-IVS-myr-GFP* |
| Figure 2 (e’) | Perineurial glia | *ifc^-/-^* mutant | *ifc-KO/ifc^js3^; GMR 85G01-GAL4/10XUAS-*  *IVS-myr-GFP* |
| Figure 3 (a) | Cortex glia | Wild type | *yw* |
| Figure 3 (b) | Cortex glia | *ifc^-/-^* mutant | *ifc^js3^/ifc-KO; repoGAL4/UAS-NLS-GFP* |
| Figure 3 (c) | Cortex glia | Pan-neuronal *ifc RNAi* | *elavGAL4/+; repoGAL80/+; UAS-ifc[RNAi]/+* |
| Figure 3 (d) | Cortex glia | Pan-glial *ifc* RNAi | *repoGAL4/UAS-ifc[RNAi]* |
| Figure 3 (e) | Cortex glia | UAS-ifc control  in *ifc^-/-^* mutant | *ifc^js3^/ifc-KO; UAS-ifc/+* |
| Figure 3 (f) | Cortex glia | Pan-neuronal *ifc*  rescue in *ifc^-/-^* mutant | *elavGAL4/+; ifc^js3^/ifc-KO repoGAL80; UAS- ifc/+* |
| Figure 3 (g) | Cortex glia | Pan-glial *ifc* rescue in *ifc^-/-^* mutant | *ifc^js3^/ifc-KO; repoGAL4/UAS-ifc* |
| Figure 3 (h) | Cortex glia | Pan-neuronal *DEGS1* rescue in *ifc^-/-^* mutant | *elavGAL4/+; ifc^js3^/ifc-KO repoGAL80; UAS- DEGS1/+* |
| Figure 3 (i) | Cortex glia | Pan-glial *DEGS1* rescue in *ifc^-/-^* mutant | *ifc^js3^/ifc-KO; repoGAL4/UAS-DEGS1* |
